# Perceptions of primary care professionals about sharing patient care with endocrinologist medium sized-city

**DOI:** 10.1186/1758-5996-7-S1-A181

**Published:** 2015-11-11

**Authors:** Andrea CBB Duvoisin, Ricardo Larroyed de Oliveira, Elisabeth Maria Nardelli de Oliveira

**Affiliations:** 1 Prefeitura Municipal de São Bento Do Sul, São Bento Do Sul, Brazil

## Background

General physicians in the Family Health Strategy assist most diabetic patients in medium sized-city according to the principles of primary health care. Although in recent yrs. there has been an increase in the number of Family Health teams, the numbers of hospitalizations for diabetes complication continue to arise in the city.

## Objective

To improve the quality of the assistance, without taking the responsibility of the primary care professionals, was conducted a case discussion workshop to enhance the ability of general practitioners in caring for diabetic patients.

## Materials and methods

The meeting had two stages: the valuation of primary care in the control of the diabetes and the role of the endocrinologist as a supporter for primary health care professionals. After the meeting was conducted a qualitative study to assess the perception of the primary care professionals about sharing patient care with the endocrinologist.

## Results

Participants included 22 primary care professionals. In the first stage of the meeting, most professionals reported difficulties and lack of technical preparation to deal with diabetic patients who require insulin, even in one per day insulin application. This lack of capacity reflected in the numerous referrals to the endocrinologist of primary health care diabetic patients. After the second stage of the meeting, with the clinical case discussion with the endocrinologist, the primary care professionals reported increased security in the diabetic patient care and reported that work together with the specialist has benefits for both the patient and for the professionals. Furthermore, reported that this support partnership has the potential to reduce the number of unnecessary referrals and improve the quality of diabetic patient care without transferring the responsability of the primary health care professionals.

## Conclusion

The participation of the endocrinologist as a support to primary health care professionals increases the general practitioner capacity to solve problems, the better use of referrals and the opening of an inter-professional communication channel, whose major beneficiary will be the diabetic patient. Advantages of a inter communication channel between the endocrinologist and professional attention to primary health.

**Figure 1:**
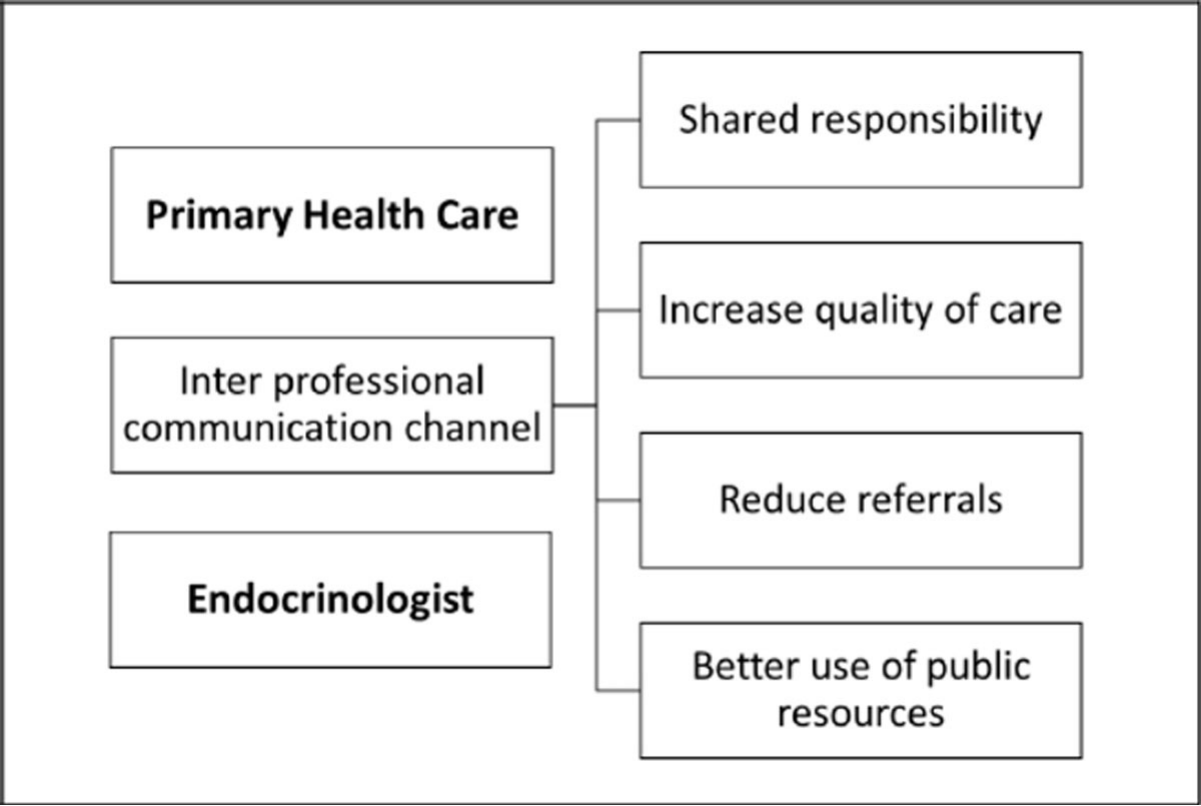
Advantages of an inter communication channel between the endocrinologist and primary health care professionals.

